# Oxidative stress markers among post-Covid-19 survivors in Central India

**DOI:** 10.6026/973206300211922

**Published:** 2025-07-31

**Authors:** Prashansa Jain, Prachi Paliwal, Jagdish C. Hundekari, Manoj Paliwal, Trupti Bajpai, Jagrati Rajput, Kushagra Mishra

**Affiliations:** 1 Sri Aurobindo Medical College and PG Institute, Indore, Madhya Pradesh, India, India; 2 Department of Biochemistry, Sri Aurobindo Medical College and PG Institute, Indore, Madhya Pradesh, India; 3 Department of Physiology, Government Medical College, Ratlam, Madhya Pradesh, India; 4 Department of Biochemistry, Government Medical College, Ratlam, Madhya Pradesh, India; 5 Department of Microbiology, Sri Aurobindo Medical College and PG Institute, Indore, Madhya Pradesh, India

**Keywords:** Covid 19, oxidative stress, post COVID-19 survivors, Superoxide dismutase (SOD), Malondialdehyde (MDA)

## Abstract

Oxidative stress is an important element in the pathophysiology of COVID-19 leading to the development of post-COVID syndrome. Therefore, it is interest to evaluate the relationship between changes in oxidative status and the persistence of long-COVID symptoms. Our, data shows that the Superoxide dismutases and Serum Malondialdehyde (MDA) levels were low in cases than controls among post-Covid-19 srvivors.

## Background:

Combating with the devastating effects of COVID-19 disease is a major challenge throughout the globe [1]. Studies have documented that SARS-CoV-2 is known to play a major role in their ability to trigger oxidative stress among the infected persons [1]. Oxidative stress is a result of imbalance between the oxidizing system and the antioxidant systems of our body, and is an important factor responsible for causing physiological and metabolic alterations [2]. COVID-19 attack provokes not only the cytokine storm but also the oxidative storm which create a havoc leading to the lethal respiratory distress [2]. Acute respiratory distress syndrome is the major cause of death among the severe Covid patients is a consequence of cytokine storm, endothelial inflammatory reactions and vascular thrombosis [3]. The complicated pathogenic pathways of COVID-19 have greatly challenged the medicinal therapy hence there is an urgent need to effectively locate the destruction pathways of COVID-19 [3]. Oxidative stress constitutes one of the signalling pathways, that intervenes with other pathogenic pathways & mediators that are responsible for pathogenicity of the disease [3]. Studies have verified the presence of oxidative stress markers in the blood samples of hospitalized patients [4]. Therefore, it is of interest to relate oxidative stress markers among post COVID-19 survivors.

## Methodology:

This was a case control study which was conducted in Indore district for a period of 2 months. A total of 60 subjects were included in the study. 30 covid-19 survivors who had history of covid-19 within past 6 months and 30 healthy controls were included in the study. Healthy subjects without any underlying condition and not having covid-19 history past 6 months were considered as control group and subjects with laboratory confirmed RT-PCR (Real Time Polymerase Chain Reaction) test for covid-19 were considered as cases. All the subjects are between 18-40 years of age. Informed consent was taken from all subjects before including them in the study. Permission from institutional ethical committee had also been taken. All the data is analysed using SPSS software with the help of institutional statistician. Pearson's correlation is done between quantitative variables. Independent t-test was used to compare means between cases and controls. Data was expressed in Mean ± SD with p value < 0.05 considered as significant. Subjects with the history of diabetes, hypertension, cancer, autoimmune, endocrine disorders, alcoholic and smokers were excluded from control and case group. The blood sample was collected in plain tube vacutainer and centrifuged at 2500 rpm for 5 minutes to separate serum. Superoxide Dismutase (SOD) and serum malondialdehyde (MDA) were estimated by Marklund & Markland method and thiobarbituric acid method respectively.

## Results:

Serum Malondialdehyde (MDA) and Superoxide dismutase (SOD) levels were estimated. The study was performed in 30 post covid survivors and 30 normal healthy volunteers as controls. The number of males in cases and control are 53% and 37% respectively. The number of Females in cases and control are 47% and 63% respectively. The mean age in cases and control are 25.63±5.23 and 21.03±1.06 respectively. Table 1 (see PDF) shows that mean serum values of SOD in cases and control are 80.67 ± 55.95 and 114.67 ± 50.63 respectively and statistically significant (p<0.05). While the mean serum values of MDA in cases were found to be low as compared to control and were found to be statistically non-significant (p>0.05). MDA levels in males and females are found to be 1.94 ± 0.95 and 1.91 ± 1.03 respectively in cases and 2.22 ± 0.92 and 1.93 ± 0.98 respectively in control. SOD levels in males and females are found to be 95 ± 66.33 and 64.29 ±36.94 respectively in cases and 130.91 ± 50.09 and 105.26 ± 49.82 respectively in control.

## Discussion:

Coronavirus disease 2019 (Covid-19) pandemic has rapidly spread around the world and encountered as a significant threat to global health [5]. Previous studies have mentioned several metabolic alterations in patients during covid infection. COVID-patients suffer from alterations in kidney function, resulting in elevated proteinuria, hematuria [6]. Many authors have shown the association between oxidative stress and the pathogenesis of COVID-19 [3]. High serum level of oxidative stress and inflammatory markers and a low serum level of antioxidants were seen patients admitted to intensive care unit. In our study we found that serum values of MDA in cases were found to be low as compared to control and were found to be statistically non-significant (p>0.05). The reason for low serum MDA levels in cases as compared to controls in our study may be due to less infectivity of virus strain as in that period omicron strain was prevalent. Oxidative stress, which encompasses reductive stress, can result in numerous harmful effects on the body, such as inflammation, tissue injury, and cellular apoptosis. Furthermore, it is implicated in complications affecting other bodily systems, especially the cardiovascular system, resulting in issues like myocarditis, thrombosis, and arrhythmias. The mean serum values of SOD in cases were found to be low as compared to control and were found to be statistically significant (p<0.05). Mehri *et al.* studied a total of 48 persons (24 with Covid-19 and 24 controls) and found that Serum MDA and TOS were significantly increased in Covid-19 patients [7]. The serum level of SOD was significantly increased in case group 25.22 ± 5.75 and 34.89 ± 7 (U/mL) in comparison to control group 19.11 ± 4.72 (U/mL); p <0.001.

## Conclusion:

The Superoxide dismutases and Serum Malondialdehyde (MDA) levels were low in cases than controls among post COVID-19 survivors.
